# The impact of grandparenting on mental health among rural middle-aged and older adults in China: exploring the role of children’s support

**DOI:** 10.3389/fpsyt.2024.1365271

**Published:** 2024-03-27

**Authors:** You-Hua Wang, Xiao-Liang Hu, Yue Li

**Affiliations:** ^1^ College of State Governance, Southwest University, Chongqing, China; ^2^ Department of Cardiology, The Ninth People’s Hospital of Chongqing, Chonqing, China

**Keywords:** grandparenting, mental health, children’s support, rural middle-aged and older adults, China

## Abstract

**Objectives:**

In the rural regions of China, characterized by a pronounced aging demographic and limited resources, a substantial proportion of middle-aged and older adults engage in grandparenting roles. Yet, the literature lacks consistent evidence regarding the effects of grandparenting on the mental health of this cohort. Accordingly, this study aimed to explore the impact of grandparenting on the mental health of rural middle-aged and older adults, as well as the underlying mechanisms.

**Methods:**

This analysis encompassed 10,881 middle-aged and older adults, utilizing data from the 2018 Harmonized China Health and Retirement Longitudinal Study (CHARLS). The mental health of participants was assessed using the Center for Epidemiological Studies Depression-10 (CESD-10) scale, while support from children was categorized into financial and emotional types. The study employed logistic and OLS regression models to identify the mediating role of child support and utilized the Karlson-Holm-Breen (KHB) method for decomposing this mediating effect.

**Results:**

The findings demonstrated that grandparenting had a significant negative impact on depression among rural middle-aged and older adults. Furthermore, children’s support played a vital role in mediating this relationship, accounting for approximately one-third of the overall influence. Moreover, the decomposition analysis revealed that both emotional and economic support from adult children equally contributed to the declination of depression among rural middle-aged and older adults.

**Conclusion:**

Grandparenting significantly enhances mental well-being in rural middle-aged and older adults, with the support from adult children serving as a vital pathway for this positive impact. Both economic and emotional assistance from children hold equal importance in this dynamic. It underscores the necessity of fortifying the family support system to amplify the support provided by children, which in turn could significantly enhance the mental health of rural middle-aged and older adults.

## 1 Introduction

In today’s rapidly evolving social landscape, marked by intense competition and increasing mobility, the role of middle-aged and older adults as caregivers for their grandchildren has become increasingly prominent worldwide (1). This trend is particularly notable in China, where an astonishing 73% of middle-aged and older adults are engaged in various levels of grandchild care, especially in rural areas affected by significant migration of the younger population from rural to urban (2). While this grandparenting role offers relief to adult children, it concurrently poses mental health challenges for the caregivers themselves (3). Understanding these challenges is crucial, as they have implications not only for the caregivers’ well-being but also for the broader family dynamics and societal health in these rapidly changing communities.

The ‘Healthy China 2030’ plan, launched by the Chinese government in 2016, emphasized the importance of mental health and care services for middle-aged and older adults. In light of this policy, the relationship between grandparenting and mental health has garnered more attention. However, existing research in Chinese contexts is still controversial, which may result from diverse theoretical frameworks and varied empirical findings in these studies (4–6). These discrepancies highlight the complexity of the issue and necessitate further investigations to validate and refine these theories.

Influenced by China’s dual social structure, compared to their urban counterparts, the formal social security for rural middle-aged and older adults is significantly inadequate (7). Additionally, under the long-term influence of China’s traditional Confucian culture, which emphasizes the importance of family, their welfare depends more on support from within the family, especially from adult children (8). Although many scholars have previously identified the significant impact of children’s support on the welfare of rural middle-aged and older adults, in the context of long-term material and financial scarcity, scholars have primarily focused on economic support (9). Meanwhile, with grandparenting becoming increasingly common, few scholars have explored whether children’s support plays a role in the relationship between grandparenting and the mental health of the caregivers.

Therefore, this study aims to examine the relationship between grandparenting and the mental health of middle-aged and older adults in rural China, and the role of children’s support in this relationship. The main contributions of this study include: first, based on nationally representative data and rigorous statistical analysis strategies, this study provides new insights into previously contested research conclusions and helps to deepen understanding of the mechanisms of grandparenting. Second, the study includes both economic and emotional support from children, facilitating a comprehensive understanding of the role and the degree of children’s support. Lastly, this study not only aids in addressing the mental health challenges faced by middle-aged and older adults in rural China effectively but also serves as a reference for other countries or regions experiencing rapid population aging and middle- to low-income challenges in promoting mental health.

## 2 Literature review

### 2.1 Grandparenting and mental health

Role strain theory suggests that individuals juggling multiple roles, particularly when they lack adequate resources, often struggle to balance or fulfill the demands of these roles. This struggle can lead to stress related to role strain, significantly affecting their physical and mental health (10). Middle-aged and older adults involved in grandparenting encounter distinctive challenges as they assume additional roles as educators, caregivers, and safety supervisors for their grandchildren. These extra duties can lead to both tangible and intangible stressors, potentially increasing the risk of depression among caregivers (11), reducing life satisfaction, and making them prone to anxiety (12).

In contrast, role expansion theory presents an alternative viewpoint, asserting that assuming multiple roles can provide individuals with additional resources, social relationships, and support, thereby enhancing their physical and mental well-being. Similarly, social participation theory suggests that engaging in social interaction and productive activities contributes to the mental health of caregivers (13). Grandparenting, as a significant avenue for productive and social engagement among middle-aged and older adults (14), holds the potential to improve their mental health. Empirical studies have indicated that long-term caregiving among older adults is associated with better self-reported health and lower levels of depression (15). Even highly demanding caregiving roles demonstrate a pronounced inhibitory effect (5, 16). Conversely, the discontinuation of grandparenting has been associated with adverse mental health outcomes in middle-aged and older adults, with these effects progressively intensifying over time (17).

Scholars have also analyzed possible reasons why grandparenting directly contributes to mental health. First, grandparenting contributes to the well-being of the family unit by instilling caregivers with a sense of purpose and accomplishment, reinforcing parental authority, and enhancing middle-aged and older adults’ perceived efficacy (18). Second, interacting with grandchildren brings joy to grandparents (19) and compensates for the potential lack of emotional support from their adult children, thereby reducing feelings of loneliness and bolstering overall life satisfaction (20, 21). Third, assuming the role of grandchild caregiver provides middle-aged and older adults with positive role experiences, fosters more positive perceptions of their own aging process, and improves cognitive function (22).

### 2.2 The mediating effect of children’s support

The stress-buffering model suggests that in high-stress situations, a strong social support network can provide resources to help individuals alleviate stress responses, thereby reducing the impact of stress on mental health (23, 24). Social support acts as a “buffer,” functioning in two main ways: direct effects, where support is universally provided in all situations, enhancing individuals’ sense of well-being and mental health; and indirect effects, where it operates in specific high-stress situations, by reducing perceived stress or increasing effective coping strategies to protect mental health (25).

Rural middle-aged and older adults engaged in grandparental caregiving face increased stress in caregiving, education and safety, compared to their counterparts without these responsibilities. At this time, external support becomes crucial. Such support can enhance the self-coping abilities of rural middle-aged and older adults undertaking grandparental caregiving and mitigate the adverse impacts of stressful events, thus making the harmful nature and severity of stress events seem less significant (26, 27).

Among various forms of support, support from adult children plays a key role. Influenced by traditional Confucian culture, rural middle-aged and older adults often place a high value on filial piety. Support from children, seen as a direct manifestation of filial piety, can create a sense of care and respect for rural middle-aged and older adults, meeting their psychological expectations and improving their sense of hope and mental well-being. Moreover, in the context of long-term resource scarcity in rural China, economic or emotional support from children can effectively alleviate the stress associated with grandparenting, reduce related burdens and tensions, promote intergenerational communication, enhance grandparents’ subjective well-being and life satisfaction (28, 29), and protect the mental welfare of the caregivers (30, 31).

### 2.3 The present study

Influenced by the long-standing dual social structure in China, where urban and rural areas have markedly different social welfare systems, older adults in rural areas often face notably constrained resources and frequently must contribute to familial welfare to secure support. Contrasting with Western cultures and deeply influenced by Confucian ethos, the older adults in rural China prioritize family continuity, commonly perceiving the provision of care to descendants as an inherent obligation. Despite ongoing discourse on the nexus between grandparenting and mental health, this study inclines towards the perspective that grandparental caregiving exerts a beneficial effect on the mental well-being of caregivers. Nevertheless, this assumption necessitates further substantiation using more stringent methodologies, and the underlying mechanisms warrant additional investigation. Furthermore, preceding research has often conceptualized children’s support as a monolithic construct, neglecting the diverse influences and ramifications of varying forms of support.

To bridge these research lacunae, the present study seeks to examine the interplay between grandparenting and mental health among middle-aged and older adults in rural China, addressing the mediating role of children’s support in this dynamic.

Hypothesis 1: Grandparenting has a significant negative effect on the depression of middle-aged and older adults in rural China.

Hypothesis 2a: The economic support of children significantly mediates the relationship between grandparenting and the depression of middle-aged and older adults in rural China.

Hypothesis 2b: The emotional support of children significantly mediates the relationship between grandparenting and the depression of middle-aged and older adults in rural China.

## 3 Methods

### 3.1 Data source

This study utilized data from the China Health and Retirement Longitudinal Study (CHARLS), a collaborative effort between Peking University and the Center for Chinese Social Survey. To ensure representativeness, the CHARLS survey was designed to include data from 28 provinces, municipalities, and autonomous regions in mainland China. For accurate sampling, a multistage probability-proportional-to-size (PPS) random sampling method was employed, supported by the use of CHARLS-GIS electronic mapping software. The survey collected a wide range of information, including demographic details, family structure and support, health status, healthcare utilization and insurance, as well as work and retirement history.

To facilitate cross-national and cross-regional comparative research on the aging process, the Gateway to Global Aging Data implemented a rigorous data cleaning process adhering to internationally accepted standards. This cleaning process followed uniform criteria, employed meticulous procedures and scientific coding, and resulted in a high-quality dataset with minimal missing values. In this study, we utilized the Harmonized CHARLS dataset, which was issued in 2018 by the designated platform. After removing individuals with missing values in relevant variables, a total of 10,881 individuals aged 45 or older were included in the final analysis.

### 3.2 Variable selection

#### 3.2.1 Dependent variable

Mental health status in China was assessed using the Center for Epidemiologic Studies Depression Scale (CESD-10). This scale measures a person’s depressive status by asking them about their behavior and feelings over the past week. The higher the total score of the scale, the more severe the depression and the more mentally unhealthy it is. The scale has been proven effective in the Chinese context and has been widely used (32). In this study, Cronbach’s alpha of this scale was 0.8137, indicating that the scale content had high internal consistency. The ten items of the scale include: (1) feeling ‘troubled’ by minor issues; (2) experiencing difficulty in ‘concentrating on tasks’; (3) feeling ‘depressed’; (4) perceiving that ‘doing anything is very difficult’; (5) feeling ‘hopeful for the future’; (6) experiencing ‘fear’; (7) having ‘poor sleep’; (8) feeling ‘happy’; (9) experiencing ‘loneliness’; and (10) feeling ‘unable to continue life’. Each item offers four response options: ‘not at all’ or ‘very well’, ‘not much’, ‘sometimes or half the time’, and ‘most of the time’. These response options are assigned scores ranging from 0 to 3. However, the items assessing ‘feeling hopeful for the future’ and ‘feeling happy’ were scored in reverse. Consequently, these two items were recoded to align with the scoring of the other items. By summing the scores of all items, the total scale score was considered a continuous variable, ranging from 0 to 30, with higher scores indicating greater levels of poor mental health.

#### 3.2.2 Independent variable

The grandparenting variable was assessed using a single-item question in this study. Participants were asked, “Have you or your spouse provided care for your grandchildren in the past year?” Respondents who answered ‘yes’ were assigned a value of 1, indicating their engagement in grandparenting. Conversely, those who answered ‘no’ were assigned a value of 0, indicating the absence of grandparenting involvement. Therefore, the independent variable is a binary variable.

#### 3.2.3 Mediating variable

The concept of children’s support encompassed both economic and emotional aspects. Economic support was operationalized as the monetary assistance received by the respondent or their spouse from their adult children in the preceding year. So, the financial support from children is a continuous variable. To reduce skewness in the distribution, the economic support from children has been log-transformed.

Regarding emotional support, the survey included inquiries about various modes of weekly communication between respondents or their spouses and their children, such as face-to-face interactions, mobile phone calls, text messages, or emails. Participants were asked whether they engaged in at least one form of communication with their children on a weekly basis. A value of 1 was assigned if respondents indicated regular weekly communication, signifying the presence of emotional support from their children. Conversely, a value of 0 was assigned if there was no regular weekly communication, indicating the absence of emotional support from their children. Therefore, emotional support from children is a binary variable.

#### 3.2.4 Covariates

Sociodemographic control variables included age (years, a continuous variable), gender (0 = female, 1 = male), marital status (0 = no spouse, 1 = has a spouse), living arrangement (0 = living children, but none co-reside nor live in the same city or county as the respondent; 1 = any child co-resides or any non-co-residing child lives in the same city or county as the respondent), social participation (0 = does not participate in social groups or activities; 1 = participates in at least one social activity), individual income (log transformation applied to reduce skewness), education level (1 = low, 2 = middle, 3 = high), and self-rated health (1 = very good, 2 = good, 3 = average, 4 = poor, 5 = very poor). These variables were included as control measures to account for potential confounding factors in the analysis.

Among the above control variables, age and individual income are continuous variables; gender, marital status, living arrangement, social participation, education level and self-rated health are either ordinal or two-state scales.

### 3.3 Analysis strategies

The analysis in this study was divided into two main sections to align with the research objectives. The first section aimed to examine the relationship between grandparenting, mental health, and children’s support through the following sequential steps: Step 1 investigated the impact of grandparenting on mental health among rural middle-aged and older adults. Step 2 examined whether grandparenting facilitated children’s support. For emotional support, a logit model was employed, while an ordinary least squares (OLS) model was used for economic support. Step 3 entailed a comprehensive analysis to assess the combined effects of grandparenting and children’s support on mental health. Additionally, the mediating effect was tentatively assessed by observing significant changes in the coefficient of the dependent variable before and after the introduction of children’s support as the mediating variable in the equation. The primary regression models utilized in this study are as follows:


(1) $$\text{Y}=\;\alpha_{R}\;+\;\beta_{R}X\;+\;\delta_{R}C\;+\;\varepsilon$$

(2) $$\text{Y}=\;\alpha_{F}\;+\;\beta_{F}X\;+\;\gamma_{F}Z\;+\;\delta_{F}C\;+\;\epsilon$$

In the regression models, Y represents the dependent variable (mental health), X represents the independent variable (grandparenting), Z represents the mediating variable (children’s support), and C represents the control variables. The constant terms and coefficients of the independent variables and control variables before the addition of mediating variables are denoted as $\alpha_{R}$ , $\beta_{R}\;$ and $\delta_{R}$ , respectively (See Equation 1). After incorporating mediator variables, $\alpha_{F}$ , $\beta_{F}$ , $\gamma_{F}$ and $\delta_{F}$ represent the coefficients of the constant term, independent variable, mediator variable, and control variable, respectively (See Equation 2).

The second section of this study focuses on utilizing the Karlson-Holm-Breen (KHB) model, as proposed by Karlson et al. (33), to examine the potential mediating role of children’s support between grandparenting and mental health. This model allows for the effective estimation of total, direct, and indirect effects. Importantly, the KHB method also enables the calculation of decomposition proportions for multiple mediating variables in direct effect simultaneously.

This study’s statistical analysis was conducted using Stata 18. To detect autocorrelation among variables, we used robust standard errors to correct for the standard errors of the coefficient estimates.

## 4 Results

### 4.1 Descriptive analysis

**Table 1** displays the findings related to the mental health status of rural middle-aged and older adults. The mean score on the depression scale was 9.09, which falls below the established criterion of 13, indicating overall good mental health (34). However, a larger standard deviation suggests greater variability in mental health within this population. Notably, 49.46% of rural middle-aged and older adults provided care for grandchildren.

**Table 1:** Basic characteristics of the participants.

Continuous variable	Mean	Standard deviation	Minimum	Maximum
Depression	9.09	6.60	0.00	30.00
Lneco_sup	6.56	3.17	0.00	12.85
Age	62.89	8.90	45.00	108.00
Lnincome	1.88	3.77	0.00	13.12

Category variable	Obs	Percentage	Cumulative percentage	
Grandparenting				
Yes	5382	49.46	49.46	
No	5499	50.54	100.00	
Emo_sup				
Yes	9671	88.88	88.88	
No	1210	11.12	100.00	
Sex				
Men	4910	45.12	45.12	
Women	5971	54.88	100.00	
Marital status				
With spouse	9279	85.28	85.28	
Without spouse	1602	14.72	100.00	
Living arrangement				
Live nearby	5849	53.75	53.75	
Live far away	5032	46.25	100.00	
Social participation				
Yes	4455	40.95	40.95	
No	6424	59.05	100.00	
Level of education				
Low	10229	94.01	94.01	
Middle	634	5.83	99.83	
High	18	0.17	100.00	
Self-reported health				
Very good	1237	11.37	11.37	
Good	1172	10.77	22.14	
Average	5220	47.97	70.11	
Poor	2529	23.24	93.36	
Very poor	723	6.64	100.00	

Lneco_sup, logarithm of children’s economic support; Emo_sup, children’s emotional support; Lnincome, logarithm of after-tax income; Obs, observation.

In **Table 1**, the mean of the log-transformed economic support from children was approximately 6.56, with a standard deviation of 3.17. 88.88% of rural middle-aged and older adults could contact their children weekly in one form. The average age of the sample was approximately 62.89 years, with males comprising 45.12% of the participants. Approximately 85.28% of the respondents reported having a spouse, and 53.75% indicated that their children resided nearby. The average after-tax income, after log transformation, was 1.88, with a standard deviation of 3.77. Additionally, approximately 94% of rural middle-aged and older adults possessed a low level of education. 22.14% of the participants rated their health as good or very good.

### 4.2 Regression analysis

Before conducting the regression analysis, a correlation coefficient matrix was created for the variables of interest to preliminarily confirm our research hypothesis (see Appendix A).

The regression results, incorporating relevant sociodemographic variables, are presented in **Table 2**. Model 1 presents the outcomes of the OLS regression analysis with depression as the dependent variable. Model 2 displays the results of the logit regression analysis with children’s emotional support as the dependent variable. Model 3 showcases the findings of the OLS regression analysis with children’s economic support as the dependent variable. Finally, Model 4 presents the comprehensive results of the OLS regression analysis with depression as the dependent variable.

**Table 2:** Regression results on grandparenting, children’s support and mental health.

Variable	Mode 1 Depression	Mode 2 Emo_sup	Mode 3 Lneco_sup	Mode 4 Depression
grandparenting	-0.306**(0.121)	0.385***(0.069)	0.285***(0.224)	-0.276**(0.121)
Emo_sup				-0.820***(0.190)
Lneco_sup				-0.010**(0.005)
Sex (ref.=Women)				
Men	-1.546^***^(0.121)	-0.169^**^(0.067)	-0.171^***^(0.062)	-1.561^***^(0.121)
Age	-0.052***(0.007)	-0.016***(0.004)	0.071***(0.004)	-0.053***(0.008)
Marital status (ref.= Without a spouse)				
With a spouse	-1.294^***^(0.173)	0.255^***^(0.093)	0.385^***^(0.089)	-1.274^***^(0.173)
Lnincome	-0.094^***^(0.016)	0.005(0.010)	-0.002(0.008)	-0.094^***^(0.016)
Education level (ref.=Low)				
Middle	-1.116^***^(0.248)	0.510^***^(0.162)	0.465^***^(0.128)	-1.079^***^(0.248)
High	-0.534(1.407)	0.924(1.049)	-1.277^*^(0.723)	-0.485(1.406)
Living arrangement (ref.= living far away)				
Living nearby	-0.156(0.115)	2.108***(0.084)	-1.250***(0.059)	-0.022(0.123)
Social activity(ref. = non-participaton)				
Participation	-0.413^***^(0.117)	0.200^***^(0.066)	0.389^***^(0.060)	-0.396^***^(0.117)
Self-reported health (ref.=Very poor)				
Very good	-8.889^***^(0.281)	0.178(0.165)	0.053(0.145)	-8.880^***^(0.281)
Good	-8.006^***^(0.283)	-0.198(0.158)	-0.032(0.146)	-8.022^***^(0.283)
Average	-6.308^***^(0.238)	-0.089(0.134)	0.030(0.122)	-6.315^***^(0.237)
Poor	-2.693^***^(0.251)	-0.298^**^(0.140)	0.027(0.129)	-2.716^***^(0.251)
Constant term	20.325***(0.596)	2.054***(0.331)	2.141***(0.306)	21.034***(0.617)
R2	0.188		0.072	0.191

Lneco_sup, logarithm of children’s economic support; Emo_sup, children’s emotional support; Lnincome, logarithm of after-tax income, presented in natural logarithm; Obs, observation; Standard errors are in parentheses; *** p< 0.01.

**Figure 1:**
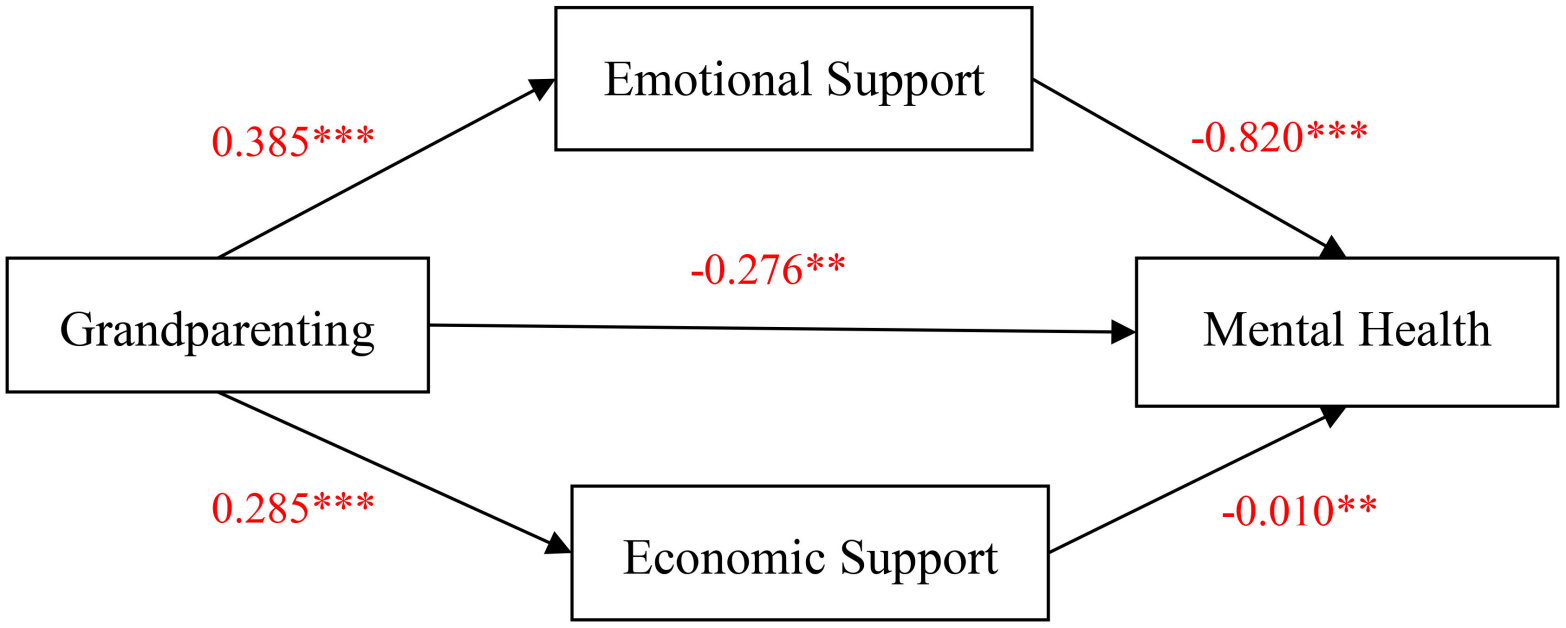
Research model and effect size. ** *p*< 0.05,*** *p*< 0.01.

Based on the findings of Model 1, which controlled for gender, marital status, educational level, living arrangements, and self-rated health, a negative association was observed between grandparenting and depression among rural middle-aged and older adults (*B* = -0.306, *p*< 0.05). This suggests that providing care for grandchildren was negatively correlated with depression among this population. Models 2 and 3 reveal significant positive associations between grandparenting and children’s emotional (*B* = 0.385, *p*< 0.01) and economic support (*B* = 0.285, *p*< 0.01), indicating that grandparenting was positively associated with emotional and economic support from children. Model 4 extends the analysis by introducing the mediating variables, children’s emotional support and children’s economic support, based on the results obtained from Model 1. The findings indicate that even after accounting for these mediating variables, grandparenting remained significantly and negatively correlated with mental health among rural middle-aged and older adults (*B* = -0.276, *p<* 0.05). Furthermore, the analysis of mediating effects revealed that children’s emotional and economic support significantly mediated the relationship between grandparenting and depression among rural middle-aged and older adults. The coefficients for emotional and financial support from adult children are -0.820 (*P*< 0.01) and -0.010 (*p*< 0.05), respectively. **Figure 1** presents the pathways of interaction among the variables of grandparenting, mental health, and the children’s support, while also displaying the corresponding specific effect values.

Consistent with previous studies (35, 36), we assessed the collinearity among the independent variables. In all four models, the variance inflation factor (VIF) was highest for self-rated health reported as “fair,” with a value of 4.33, which is below 5. This indicates that there is no multicollinearity issue among the variables in any of the models. Additionally, the presence of outliers significantly influences the model fit; hence, we log-transformed the economic support and income variables, which showed large discrepancies. Furthermore, upon inspecting the scatter plots between the independent and dependent variables, we did not observe any strong patterns or curvature.

Different treatments of variables may lead to different research conclusions. To ensure the robustness of our findings, we conducted an additional analysis by recoding the depression scale as a dichotomous variable. Scores from 0 to 10 were categorized as 0, indicating good mental health, while scores between 11 and 30 were categorized as 1, indicating poor mental health. We used the same analysis strategy as in **Table 2** for the robustness test, except that binary logistic regression was used in models 1 and 4. Although the analysis results (see Appendix B) have slight differences in coefficient, their influence direction and significance are consistent with those in **Table 2**, indicating that the main conclusions of this study have strong stability.

To gain a deeper understanding of the significance of children’s support in the mental well-being of rural middle-aged and older adults, we employed the Karlson-Holm-Breen (KHB) method to decompose the mediating effect of children’s support based on the results from **Table 2**. The findings, presented in **Table 3**, indicated that within the effects of grandparenting on mental health, children’s emotional support accounted for 18.61% and children’s economic support accounted for 13.33% of the mediating effect. Collectively, these factors amounted to a 31.39% mediating effect of children’s support on the total effect. This analysis provides further insights into the crucial role of children’s support in shaping the mental well-being of rural middle-aged and older adults.

**Table 3:** Mediating effect decomposition of children’s support.

Effect	Mode1 Emo_sup	Mode2 Lneco_sup	Mode3 Overall support
Total effect	-0.360**	-0.360**	-0.360**
Direct effect	-0.293**	-0.312**	-0.247**
Indirect effect	-0.067**	-0.048*	-0.113***
Proportion of mediating effect (%)	18.61	13.33	31.39

Emo_sup, children’s emotional support; Lneco_sup, logarithm of children’s economic support; Overall support, children’s overall support; * p< 0.1, ** p< 0.05, *** p< 0.01.

### 4.3 Endogenous issue

We employed propensity score matching (PSM) to mitigate the influence of unobserved variables on our findings. PSM significantly reduces selection bias in observational studies by balancing groups based on covariates, thereby enhancing the robustness of causal inferences. As shown in **Table 4**, slight variations were observed across different matching methods, but all results demonstrated statistical significance. This confirms the positive impact of grandparenting on the mental well-being of rural middle-aged and older adults. This robustness check reinforces the notion that grandparenting benefits the mental health of this demographic.

**Table 4:** Effect of grandparenting on mental health based on propensity score matching.

Matching strategy	Grandparenting	Non grandparenting	Difference	Standard error	T value
1:1 matching	8.808	9.432	-0.624***	0.248	-2.51
Neighbour matching	8.808	9.432	-0.624***	0.248	-2.51
Radius matching	8.808	9.180	-0.372***	0.132	-2.82
Kernel matching	8.808	9.198	-0.389***	0.139	-2.80

*** p< 0.01.

## 5 Discussion

The mental well-being of rural middle-aged and older adults in China has significant implications not only for personal and familial quality of life but also for the active aging strategy of China. This study explores the relationship between grandparenting and the mental health of middle-aged and older adults in rural China, utilizing the Harmonized CHARLS dataset. It also examines the role of children’s support within this dynamic.

### 5.1 Grandparenting and mental health

The results of this study support Hypothesis 1, indicating that grandparenting is negatively associated with depression among rural middle-aged and older adults. These results align with the findings of previous studies (37–39). For example, Yang (40) conducted a comparative study in the UK, another European country, and China, and found that grandparenting in low-income countries could mitigate depressive symptoms, unlike in high-income countries. This finding is corroborated by another study in Sri Lanka, which confirms a positive correlation between grandparenting and reduced distress (41).

In China’s dual rural-urban society, especially in resource-limited rural areas, older care predominantly depends on family support. Middle-aged and older adults engage in reciprocal care by dedicating time and energy to grandchild care, anticipating reciprocal older care from their adult children in the future (42, 43). This expectation is reinforced by traditional Confucian family values, fostering a sense of pride and self-efficacy in contributing to family continuity and development (44). Concurrently, the increase in rural-urban migration has resulted in a growing number of middle-aged and older adults living alone in rural areas. This isolation often leads to idleness, a lack of companionship, and escalating feelings of despair. In contrast, grandparenting offers these individuals a sense of purpose and emotional connection, leading to greater life satisfaction and mental health (45).

Discrepancies in the conclusions of previous studies examining the relationship between grandparenting and mental health may stem from several methodological challenges. Firstly, variations in the measurement and processing of variables can result in different statistical analyses and outcomes (46, 47). Such variations might include diverse ways of defining and quantifying mental health indicators or grandparenting. Secondly, the selection of samples plays a critical role in research consistency (48). Differences in demographic characteristics, geographic locations, or socio-economic statuses of the samples can significantly affect study results (49). Lastly, many previous studies have not adequately addressed the issue of endogeneity in variables, which can lead to misleading interpretations of the relationships between variables.

### 5.2 Children’s support as mediating role

Hypotheses 2a and 2b are validated by our mediation analysis, indicating that emotional and economic support from children has linked grandparenting and mental health among rural middle-aged and older adults in China, aligning with previous research (50, 51).

On the one hand, the results affirm that grandparenting enhances support from adult children, both emotionally and economically (52). Influenced by the traditional ethical concept of ‘reciprocating kindness’, adult children often show gratitude and increased filial piety toward parents who assist in caring for their grandchildren, reciprocating with material or emotional support. Objectively, adult children who benefit from grandparenting can invest more energy in work, potentially earning higher incomes and thus providing economic rewards (53, 54). In rural China, where traditional family ethics, particularly unconditional filial piety, are undergoing significant transformations (55), the necessity of intergenerational resource exchange becomes pronounced (21). In this context, grandparenting initiates a reciprocal flow of resources, with older adults contributing through childcare and children providing necessary economic and emotional support in response (56).

On the other hand, grandparenting strengthens familial bonds, enhancing emotional support from adult children (4). The involvement of grandchildren often amplifies interactions and emotional care from children (57). This enhanced emotional and economic support from adult children is possibly pivotal in mitigating depression risks among rural middle-aged and older adults (58, 59). In rural China, the traditional Confucian ideals of ‘raising children to care for you when you are old’ and filial piety are still accepted by most people, so older parents usually have high expectations for their children’s support (60). Children’s support meets both the material and emotional expectations of grandparents, strengthening familial ties and reinforcing intergenerational commitments (53). Moreover, despite advancements in China’s pension system, the scarcity of formal pension resources makes familial support indispensable (61). Therefore, reciprocal relationships between generations may be essential for the health of older adults.

Therefore, the effectiveness of intergenerational interactions and the security provided by adult children are crucial for diminishing depression rates among rural middle-aged and older adults in China (52, 62). Limited familial contact can lead to feelings of abandonment and emotional distress, underscoring the importance of maintaining strong family connections, in accordance with the values of filial piety (62). In addition to family intergenerational relationships, the individual demographic and social characteristics of the older adults, such as gender, education, and health status, simultaneously affect their mental health and access to mental health assistance services (63).

### 5.3 Limitations

This study has several limitations. Firstly, due to the cross-sectional design, while associations between grandparenting, children’s support, and mental health are identified, causal relationships cannot be conclusively established. It is important for future studies to employ longitudinal designs to better ascertain causation. Secondly, the independent variable, grandparenting, was operationalized as a binary variable, which fails to capture the complex dimensions of grandparenting. It notably overlooks factors such as the intensity, diversity, and frequency of caregiving activities. This oversimplification could limit the depth of our findings. Therefore, future research should aim to adopt a more nuanced and multidimensional approach to operationalizing grandparenting. Thirdly, while the CHARLS dataset provides robust data, reliance on secondary data limits the ability to customize data collection to specific research questions. This may result in potential unobserved confounding variables that could affect the results. Subsequent studies might focus on primary data collection or explore methods to mitigate the impact of unobserved variables.

## 6 Conclusions

In conclusion, our study offers substantial evidence that grandparenting is positively associated with better mental health among China’s rural middle-aged and older adults, compared to their counterparts who do not engage in such caregiving. Crucially, this study underscores the significant mediating role of adult children’s support, including both economic and emotional aspects, in this relationship. The findings emphasize the importance of nurturing support from the younger generation to promote the mental well-being of this demographic and enhance overall family welfare. These insights have important implications for policy-making and community support initiatives in rural China, underscoring the need to encourage and facilitate intergenerational support. Additionally, this study contributes to a broader understanding of the dynamics of aging in rural contexts, offering a foundation for future research in similar demographic settings.

## Data availability statement

Publicly available datasets were analyzed in this study. This data can be found here: http://charls.pku.edu.cn/en.

## Author contributions

Y-HW: Writing – review & editing, Funding acquisition, Conceptualization. X-LH: Writing – original draft, Software, Formal analysis, Data curation. YL: Writing – original draft, Supervision, Resources, Formal analysis, Conceptualization.
