# Supplementary material for: The impact of grandparenting on mental health among rural middle-aged and older adults in China: exploring the role of children’s support
Source: Front Psychiatry. 2024 Mar 27;15:1365271. doi: 10.3389/fpsyt.2024.1365271 (PMC11004320; doi:10.3389/fpsyt.2024.1365271)
Supplement: Supplementary file 1 [file Table_1.docx]

Appendix A Correlation among grandparenting, depression, and children’s support

| Variable | Depression | Grandparenting | Economic support | Emotional support |
| --- | --- | --- | --- | --- |
| Depression | 1.000 | -0.037*** | -0.032*** | -0.049*** |
| Grandparenting | -0.046*** | 1.000 | 0.015* | 0.095*** |
| Economic support | -0.029*** | 0.033*** | 1.000 | 0.038*** |
| Emotional support | -0.052*** | 0.096*** | 0.037*** | 1.000 |

Abbreviations: Economic support, Children’s economic support; Emotional support, Children’s emotional support; * *p* < 0.05; ** *p* < 0.01; *** *p* < 0.001.

Appendix B Results of robust regression analysis

| Variables | Mode 1 | Mode 2 | Mode 3 | Mode 4 |
| --- | --- | --- | --- | --- |
|  | Depression | Emotional support | Economic support | Depression |
| Grandparenting | -0.205*(0.093) | 0.394***(0.069) | 1.101***(0.223) | -0.195*(0.103) |
| Emotional support |  |  |  | -0.323*(0.189) |
| Economic support |  |  |  | -0.011*(0.009) |
| Covariates | Yes | Yes | Yes | Yes |
| Constant term | 1.966***(0.230) | 2.292***(0.340) | 1.287(1.117) | 2.195***(0.238) |

Abbreviations: Economic support, Children’s economic support; Emotional support, Children’s emotional support; Standard errors are in parentheses; * *p* < 0.05, ** *p* < 0.01, *** *p* < 0.001
